# Evaluation of the Effect of Buccolingual and Apicocoronal Positions of Dental Implants on Stress and Strain in Alveolar Bone by Finite Element Analysis

**Published:** 2018-01

**Authors:** Farhood Massoumi, Mina Taheri, Abolghasem Mohammadi, Omid Amelirad

**Affiliations:** 1 Assistant Professor, Dental Research Center, Research Institute of Dental Sciences, Department of Prosthodontics, School of Dentistry, Shahid Beheshti University of Medical Sciences, Tehran, Iran; 2 Postgraduate Student, Department of Periodontics, School of Dentistry, Tehran University of Medical Sciences, Tehran, Iran; 3 Assistant Professor, Department of Prosthodontics, School of Dentistry, Shahid Beheshti University of Medical Sciences, Tehran, Iran; 4 PhD Student, Department of Mechanical Engineering, Sharif University of Technology, Tehran, Iran

**Keywords:** Dental Implants, Dental Stress Analysis, Mechanical Stress, Finite Element Analysis

## Abstract

**Objectives:**

The position of dental implants in the alveolar bone can affect the surrounding bone from biomechanical and biological aspects. The purpose of this study was to evaluate the effect of implant position on stress and strain distribution in the surrounding bone by using finite element analysis (FEA).

**Materials and Methods:**

Thirteen computerized models of a 3.8-mm-diameter XiVE implant with the abutment and crown of a mandibular second premolar in a mandibular bone segment were designed. In the reference model, the implant was placed at the center of the alveolar ridge with its crest module located above the alveolar crest. In the other models, the implants were positioned buccally, lingually, coronally or apically by 0.5, 1 or 1.5mm. By using the ANSYS software program, a 100-N load was applied to the buccal cusp parallel to and at a 30-degree angle relative to the longitudinal axis of the fixture. The models were analyzed in terms of the distribution of stress and strain in the bone.

**Results:**

The different implant positions induced nonlinear stress and strain changes in the bone. The central, 1.5-mm apical, and 1.5-mm coronal implant positions induced high amounts of stress and strain under off-axial loads.

**Conclusions:**

Within the limitations of this study, the results showed that the stress and strain in the bone around the implant undergo small nonlinear changes with buccolingual and apicocoronal shifting of the implant and can be affected by the configuration of the implant in contact with the bone.

## INTRODUCTION

Dental implants are increasingly used due to their high survival and success rates [1]. Most implant failures occur after prosthetic loading [1] and reflect the role of biomechanical factors in the success rate of dental implants. Loosening and fracture of the abutment or occlusal screw and bone loss are among the adverse mechanical and biological effects of implant loading [2].

Marginal bone loss around dental implants is a common problem, and its amount is used as an index for determination of the success rate of implants [3]. The stress applied to the restoration and implant is eventually transferred to the surrounding bone and affects bone remodeling [4]. Some levels of stress are necessary to prevent bone atrophy, but higher amounts of stress can cause bone fracture or bone resorption [5]. Various factors can affect the amount and distribution of stress and strain in the bone such as implant-related factors including the implant’s diameter and length, screw design [6,7], and the depth of placement [8–10], bone-related factors such as alveolar bone quality, width [6] and contour [11] of the alveolar ridge, and load-related factors such as the magnitude [12] and direction of load application [8,12–16]. Several studies have evaluated various implant positions [8–10,13,17]. The apicocoronal position of the implant may be changed for various reasons, the main of which are aesthetic considerations [18,19]. A finite element analysis (FEA) by Qian et al [8] showed that the stress and strain in the bone increase around the implants placed more superficially. However, another FEA by Chou et al [9] showed that the depth of implant placement had no significant effect on the level of strain in the bone. Lee et al [17] reported that the clinical success of implants coated with hydroxyapatite was higher when placed subcrestally compared to their equicrestal positioning.

The effect of the buccolingual position of implants on the stress and strain in the surrounding bone has been limitedly evaluated, although it can be an important factor as it plays a role in the quality and quantity of the peri-implant bone and soft tissue status. Also, there is a risk of generation of cantilever forces with different buccolingual implant positions, which can affect the stress and strain in the bone [1].

Regarding the angle of load application, Qian et al [8] showed that increasing the angle of load application from 0° to 45° significantly increases the stress and strain in the bone. Huang et al [13] and Chang et al [14] also demonstrated that increasing the angle of load application is the most important factor responsible for a higher stress and strain in the surrounding bone. Considering the significant effect of the angle of load application on the stress and strain in the peri-implant bone and the presence of off-axial loads in the oral cavity exerted on teeth during the function, assessment of the effect of oblique loads on the stress and strain in the bone seems essential.

Considering the controversy on the effect of the intraosseous implant position on its success rate, this study aimed to assess the effect of dental implant position on the stress and strain in the surrounding bone by using FEA.

## MATERIALS AND METHODS

**Fig. 1:**
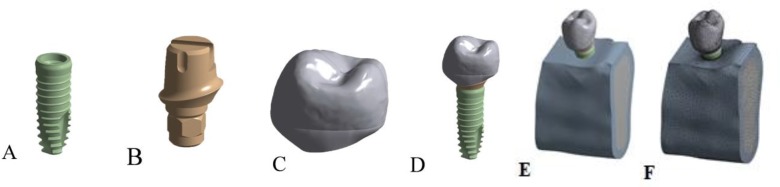
Three-dimensional (3D) finite element analysis (FEA) models of (A) implant, (B) abutment, (C) crown, (D) crown and abutment on the fixture, (E) reference model with the implant positioned at the center of the alveolar ridge, and (F) meshing of the reference model

Thirteen computerized models of a single-tooth implant with a mandibular second premolar’s crown in a segment of an edentulous mandible were designed. The scanning and initial modeling of the alveolar bone, abutment, crown, and fixture were performed according to a previous study by Sahabi et al [20] (Fig. 1). The computerized tomography (CT) images of a fully developed mandible were used for three-dimensional (3D) modeling of a mandibular bone segment. The CT data in the Digital Imaging and Communications in Medicine (DICOM) format were transferred to the Rapidform® software program (INUS Technology, Seoul, South Korea) to obtain a solid 3D model. A XiVE dental implant (Dentsply Friadent, Mannheim, Germany) with the diameter (D) of 3.8mm and length (L) of 11mm (XiVE S Plus Implant D=3.8mm/L=11mm) with a Friadent EstheticBase straight abutment (D=3.8mm/gingival height (GH)=1mm/angle (A)=0°) were digitized at high resolution using the light digitizing system (ATOS, GOM, Braunschweig, Germany). Data were recorded in the Standard Triangle Language (STL) format. In order to make an accurate model of a right mandibular second premolar, a plastic model of this tooth (Nissin Dental Products INC., Kyoto, Japan) was scanned using the computer-aided design/computer-aided manufacturing (CAD/CAM) system (Tizian CAD/CAM system, Schütz Dental GmbH, Rosbach, Germany), and data were acquired in the STL format. The data were converted to the Standard for the Exchange of Product Data (STEP) format using the ScanTo3D option (SolidWorks Crop., Concord, Massachusetts, USA) to become readable by the SolidWorks® 2008 software program (SolidWorks Crop., Concord, Massachusetts, USA). In the SolidWorks® software program, the coronal anatomy was adapted to the abutment collar to create a normal cervical anatomy of an implant-supported crown. The 3.8-mm-diameter abutment model was shortened to the height of 4mm above the collar, and a circumferential bevel with a 0.4-mm width was created at the occlusal end of the abutment to prevent stress accumulation in this area. Next, the crown, the modified abutment, and the fixture were assembled. The thickness of the crown was at least 1.5mm in all areas, and a 25-μm cement space was considered between the abutment and crown [21–23]. All the gaps between the abutment and crown were filled by the cement. In the reference model, the implant was placed at the center of the buccolingual width of the alveolar ridge. The longitudinal axis of the fixture was perpendicular to the alveolar crest such that the crest module and the high-polished area of the fixture were located above the alveolar ridge. In the subcrestal models (n=3), the smooth area of the fixture, located beneath the alveolar crest, was considered not in contact with the bone. In the model with the implant positioned 1mm lingual to the center of the alveolar ridge, the apical part of the implant was in contact with the lingual cortical plate. This contact was even greater in the model with the implant placed 1.5mm lingual to the center of the alveolar ridge (but not protruding out of the lingual cortical plate leading to cortical plate perforation).

Thus, different apicocoronal positions of 1.5, 1 and 0.5mm below the alveolar crest (subcrestal position, n=3) and 0.5, 1 and 1.5mm above the alveolar crest (supracrestal position, n=3), and different buccolingual positions of 0.5, 1 and 1.5mm lingual (n=3) and 0.5, 1 and 1.5mm buccal to the center of the alveolar crest (n=3) were designed.

The ANSYS software program (version 15.0.1, ANSYS Inc., Pennsylvania, USA) was used for FEA. All the materials were considered homogeneous, isotropic and linearly elastic, and the contacts were considered complete. Table 1 shows the physical properties of the components [12,22,24,25]. The meshing of the models was done using 10-node tetrahedral elements with an approximate size of 0.1-1mm around the implant neck. Smaller elements were used in the peri-implant bone. Each model included 520,000 elements and 750,000 nodes. All the contacts were considered to be of the bonded type, where the displacement components of the surfaces in contact are tied up together so that there is no separation or penetration. The models were considered fixed in the bone sections. A 100-N load was applied to the buccal cusp tip of the crown along the longitudinal axis of the fixture and also at a 30-degree angle (buccolingually) relative to the longitudinal axis of the fixture. The analysis was performed separately for each load. The location and magnitude of the maximum and minimum principal stress and strain in the cortical and cancellous bone were determined. The stress and strain patterns and their magnitude were compared in different models.

**Table 1.** Physical properties of the components in the finite element analysis (FEA)

**Material**	**Young’s modulus (MPa)**	**Poisson’s ratio**
**Cortical bone**	15000	0.3
**Cancellous bone**	1500	0.3
**Titanium alloy**	110000	0.33
**Casting gold alloy**	91000	0.33
**Polycarboxylate cement**	5110	0.35

## RESULTS

**Fig. 2:**
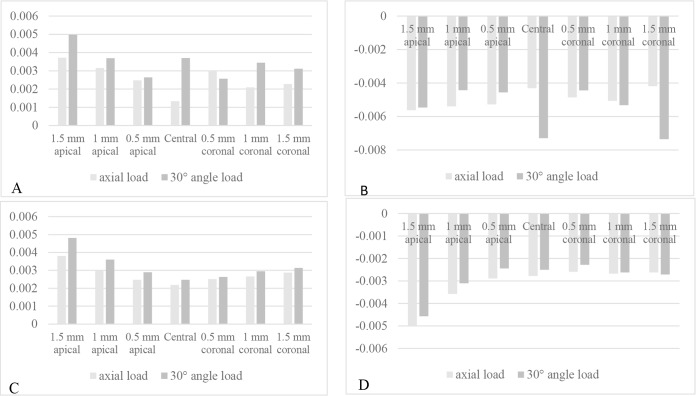
Apicocoronal positions. (A) Maximum principal strain in the cortical bone. (B) Minimum principal strain in the cortical bone. (C) Maximum principal strain in the cancellous bone. (D) Minimum principal strain in the cancellous bone. The related stress values follow a similar pattern

**Fig. 3:**
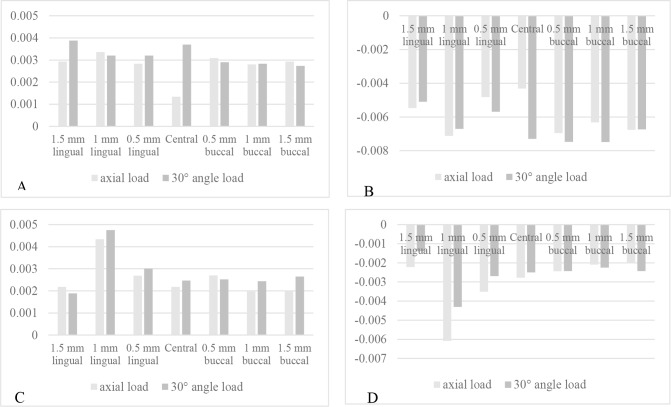
Buccolingual positions. (A) Maximum principal strain in the cortical bone. (B) Minimum principal strain in the cortical bone. (C) Maximum principal strain in the cancellous bone. (D) Minimum principal strain in the cancellous bone. The related stress values follow a similar pattern.

**Fig. 4:**
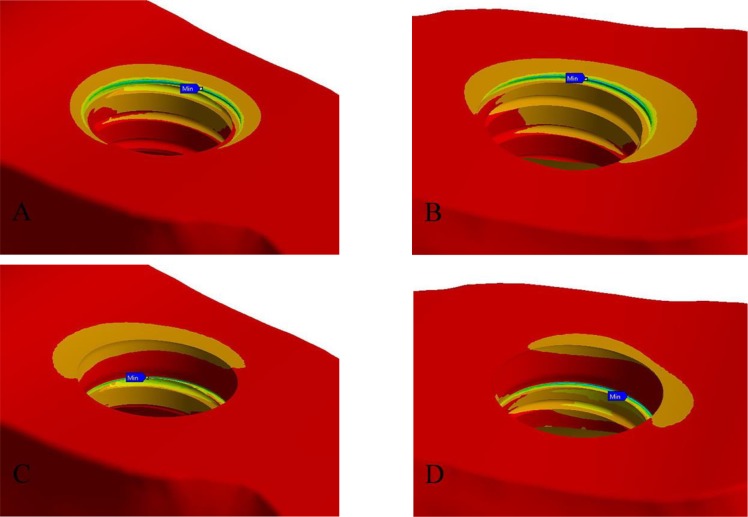
Site of maximum compressive stress; under the axial load, this site was at the buccal side of the implant, while under the off-axial load, this site was at the lingual side of the implant. (A) and (B) The reference model under axial and off-axial loads, respectively. (C) and (D) The model with an implant positioned 1-mm apically under axial and off-axial loads, respectively

Figures 2 and 3 show the maximum and minimum principal stress and strain in different models. The compressive stress and strain in the cortical bone were concentrated at the most coronal contact between the implant and bone at the buccal surfaces of the implants under the axial load, and at the lingual surfaces of the implants under the off-axial load (Fig. 4). Under the axial load, the concentration of the tensile stress and strain in the cortical bone occurred at the first bone-implant contact at the lingual surfaces of the implants in all the models except for the models with an apical displacement, where the concentration of the tensile stress and strain occurred at the buccal surfaces of the implants. Under the off-axial load, the concentration of the tensile stress and strain in the cortical bone occurred at the first bone-implant contact at the buccal aspect of the implants, except for the model with an implant positioned 0.5-mm apically in which the concentration of the tensile stress occurred at the lingual surface of the implant. There was no linear relationship between the amounts of stress and strain in the bone and different buccolingual or apicocoronal implant positions. In the reference model, both the tensile and compressive stresses in the cortical bone increased by 50 MPa under the off-axial load, while the tensile and compressive strains in the cortical bone increased by 0.0024 and 0.003 units, respectively (compared to the axial load). However, in the other models, the obtained values under the axial and off-axial loads were close, except for the model with an implant positioned 1.5mm coronally, in which the tensile and compressive stresses in the cortical bone increased by 15 and 45 MPa, respectively, while the compressive strain increased by more than 0.003 units.

In most models, the highest amounts of stress and strain in the cancellous bone were recorded around the tip of the implant threads, close to the apex, at the lingual or mesial sides of the implant. However, in the reference model and in the models in which the implants had been positioned 1.5mm coronally, 1.5mm buccally and 0.5mm buccally under the axial and off-axial loads, the maximum tensile stress was noted right below the crest of the cortical bone at the lingual and buccal surfaces of the implants, respectively. Among the apicocoronally-shifted implants, the values recorded for the cancellous bone in the models with implants positioned 1.5-mm apically were greater than the values in the other models. In the buccolingually-shifted implants, the cancellous bone showed the highest stress and strain values in the model with an implant positioned 1mm lingual to the center of the alveolar ridge.

## DISCUSSION

The roles of implant design and dimension [6,7], bone quality [9,10,13,26,27] and implant position [8–10,13,17,28,29] in the success of dental implants have been previously evaluated from biological and biomechanical aspects. The main focus has been placed on the submerged and equicrestal implants. In this study, we assessed the effect of different apicocoronal and buccolingual implant positions on the stress and strain in the surrounding bone.

In terms of the apicocoronal position, the reference model under the axial load yielded the best results (the smallest stress and strain values); however, under the off-axial load (30°), this model showed a great increase in the stress and strain such that except for the 1.5-mm apical positioning of the implant with higher tensile stress and strain values and the 1.5-mm coronal positioning of the implant with almost the same levels of compressive stress and strain, the reference model represented the worst positioning among all the models. There does not seem to be a clinical justification for this increase of stress and strain in the reference model. However, optimal results in the reference model under the axial load in our study were in agreement with those of the study by Huang et al [13] in which the equicrestal position yielded the least amount of compressive stress, and with those of the study by Rismanchian et al [24] in which placing the implant 0.1mm subcrestally yielded the lowest von Mises stress values. Huang et al [13] showed that a change in the angle of the load applied to the implant (from 0° to 45°) did not make the equicrestal position more unfavorable than the other positions. In the study by Rismanchian et al [24], a change in the angle of the load from axial to 15° mainly increased the stress in the supracrestal positions and subcrestal positions deeper than 0.8mm, but the curve of alterations in the stress at different positions was the same under axial and off-axial loads.

Qian et al [8] and Chu et al [10] found that by an increase in the depth of insertion, the stress and strain values decreased compared to the equicrestal position. The same was true in our study under the off-axial load. However, the 1.5-mm apical positioning did not seem suitable, while the 0.5- or 1-mm apical and 0.5- or 1-mm coronal positions yielded the best results under the off-axial load. In an FEA study by León et al [30], the supracrestal position of the implant induced higher compressive stress and lower tensile stress values compared to the subcrestal position. However, since the strain values in none of the two models were within the overloading range, the subcrestal model was suggested for implant placement due to aesthetic and prosthetic advantages [30].

It should be noted that due to the presence of evidence [31–34] supporting no attachment between the smooth or high-polished implant surfaces and bone, this part of the fixture was considered not bonded to the bone in our study. Thus, when the implant was placed apically, it further contacted the cancellous bone instead of the cortical bone. Since the cancellous bone has a lower modulus of elasticity than the cortical bone [35], the magnitude of stress and strain will be increased in contact with titanium [36]. This can explain the high stress and strain values in the model with an implant positioned 1.5-mm apically.

Among the models with different buccolingual implant positions, the reference model showed the least amounts of almost all the analyzed parameters under the axial load but not under the off-axial load. This may be due to the greatest bone thickness on both buccal and lingual sides of the implant as it is shown that the stress is reduced with increasing bone thickness [37,38]. However, a severe increase in the compressive and tensile stress and strain in the cancellous bone in the model with an implant placed 1mm lingually may be due to the unique geometry of the model in this position; involvement of the apical implant threads with the cortical bone is such that the volume of the cancellous bone remaining between the implant and cortical plate is very small, and thus, it undergoes high levels of stress and strain. One may consider this increase in the stress and strain to be due to the bicortical engagement of the implant with the bone; however, in the study by Chang et al [14], the monocortically- and bicortically-engaged implants did not show any significant difference in stress and strain values.

Another example revealing the effect of geometry on the distribution of stress and strain was the model with an implant placed 1.5mm buccal to the center of the alveolar ridge, where one thread was located just beneath the cortical bone, and the concentration of stress in the cancellous bone also was detected to be at this site.

Overall, it can be stated that in the cortical bone, the maximum concentration of compressive and tensile stress and strain was noted at the site of the first bone-implant contact. This was in line with the results of other related studies [8,9,30] and with the clinical results showing a greater marginal bone loss [28,30,39]. In all the models, the maximum stress and strain in the cancellous bone were concentrated around the tip of the apical threads of the implant. This distribution pattern was similar to that observed by Qian et al [8] and Hsu et al [40].

The stress and strain values in most specimens did not significantly vary by changing the vertical angle of load application from 0° to 30°. This may be due to the use of crowned models in our study since the axial load in such situation (although parallel to the longitudinal axis of the fixture) was eccentric (buccally relative to the center of the implant). This means that the crown and implant are subjected to cantilever forces. The role of cantilever forces in increasing the stress and strain in the bone has been previously documented [41–43]. According to Frost [5], strains of 2500 to 4000 microstrains induce remodeling and increase the density and physiological hypertrophy of the bone, while values higher than 4000 microstrains are pathological for the bone since they create internal cracks, which cannot be repaired by natural bone remodeling [5]. Pattin et al [44] showed that the maximum physiological tensile and compressive strains in the bone are equal to 2500 and 4000 microstrains, respectively. They discussed that a compressive stress of less than 40MPa was physiological for the bone, while a 50-MPa compressive stress (3600 microstrains) is the critical threshold at which bone resorption occurs in higher volumes [44]. Thus, the maximum stress and strain values in our study were pathological in most models. However, a very small volume of bone was subjected to such high values, while in other areas, the peri-implant bone was under physiological levels of stress and strain.

A noteworthy issue in the clinical setting is that different buccolingual and apicocoronal implant positions require some changes in the shape and contour of the prosthesis to obtain correct proximal and occlusal contacts with the adjacent and opposing teeth. However, in the current study, we simply used the same coronal geometry in all the models. More accurate results can be obtained if the changes in the crown contour are also taken into account. Nonetheless, modeling of the crown and cement was one of the strong points of our study as it has been neglected in several previous studies.

## CONCLUSION

Within the limitations of this study, the results showed that the stress and strain distribution in the peri-implant alveolar bone undergoes small nonlinear changes with buccolingual and apicocoronal shifting of the implant and can be affected by the configuration of the implant in contact with the bone.
